# Hyperprogressive Disease in an Advanced Stage Colon Cancer Patient on Pembrolizumab: A Case Report

**DOI:** 10.7759/cureus.7764

**Published:** 2020-04-21

**Authors:** Kok Hoe Chan, Saraswathi Lakkasani, Amr Ramahi, Hamid S Shaaban

**Affiliations:** 1 Internal Medicine, Saint Michael's Medical Center, Newark, USA; 2 Hematology and Oncology, Saint Michael's Medical Center, Newark, USA

**Keywords:** hyperprogressive, colon cancer, anti-pd1, pembrolizumab

## Abstract

The emergence of immune checkpoint inhibitors (ICIs) in recent years has transformed the landscape of the management of solid tumors. The advancement of immunotherapy has resulted in a brand new set of adverse outcomes not previously seen in classical chemotherapy. One such adverse effect has been termed as hyperprogressive disease (HPD), a phenomenon characterized by rapid tumor progression, which often leads to devastating outcomes. In this report, we present a unique case of a 48-year-old African American female who initially presented with abdominal pain, fatigue, and weight loss. Subsequent CT scan showed extensive irregular wall and luminal narrowing with an eccentric mass and adenopathy along the portacaval space. Tumor markers were found to be elevated and genetic testing was done. The patient was diagnosed with stage IIIC colon cancer with K-RAS wild type, associated with Lynch syndrome. The patient underwent surgical resection, chemotherapy, and targeted therapy for progressive/stage IV disease. In light of the progression of the disease, pembrolizumab was introduced into the treatment regimen. One month after the treatment, a repeat CT scan showed enlargement of the metastatic lesion with almost double the size. The progression of the disease was so rapid and, ultimately, pembrolizumab administration was withheld and the patient passed away after about two months on pembrolizumab. To our knowledge, this is one of the few cases of HPD reported in patients with advanced colon cancer, particularly in one with Lynch syndrome. Further studies are warranted to understand why some individuals benefit from immunotherapy, whereas others experience grave outcomes.

## Introduction

In recent years, immune checkpoint inhibitors (ICIs) such as anti-programmed death 1 (anti-PD-1) and anti-programmed death-ligand 1 (anti-PD-L1) have changed the landscape of the management of patients with advanced solid tumors, especially non-small cell lung cancers (NSCLC) and melanoma. The advent of immunotherapy has also resulted in a brand new set of adverse outcomes and tumor responses not previously seen in classical chemotherapy. One such adverse effect has been termed as hyperprogressive disease (HPD) [1].

Champiat et al. were the first to describe a unique phenomenon of paradoxical acceleration of tumor growth in cancer patients treated with ICIs. This unique phenomenon is named as HPD [1]. HPD has been reported in a wide variety of cases including melanoma, NSCLC, lymphoma, ovarian malignancies, urothelial cancer, and colorectal cancer (albeit rarely). There are a few suggested predictors of HPD in solid tumors treated with ICIs, and the incidence observed was about 2.6-13.8% across three retrospective analyses [1-3]. Lynch syndrome, on the other hand, is the most common inherited autosomal dominant disorder, characterized by microsatellite instability with the germline mutation or loss of deletion of DNA mismatch repair genes [4]. Herein, we report a case of HPD after treatment with pembrolizumab in a patient who progressed from stage III to stage IV colon cancer, subsequently diagnosed with Lynch syndrome, and failed the standard regimen. To our knowledge, this is one of the very few case reports on HPD in advanced stage colon cancer treated with pembrolizumab. We believe our report contributes to the limited literature on HPD in advanced stage colon cancers, particularly those with Lynch syndrome.

## Case presentation

**Figure 1:**
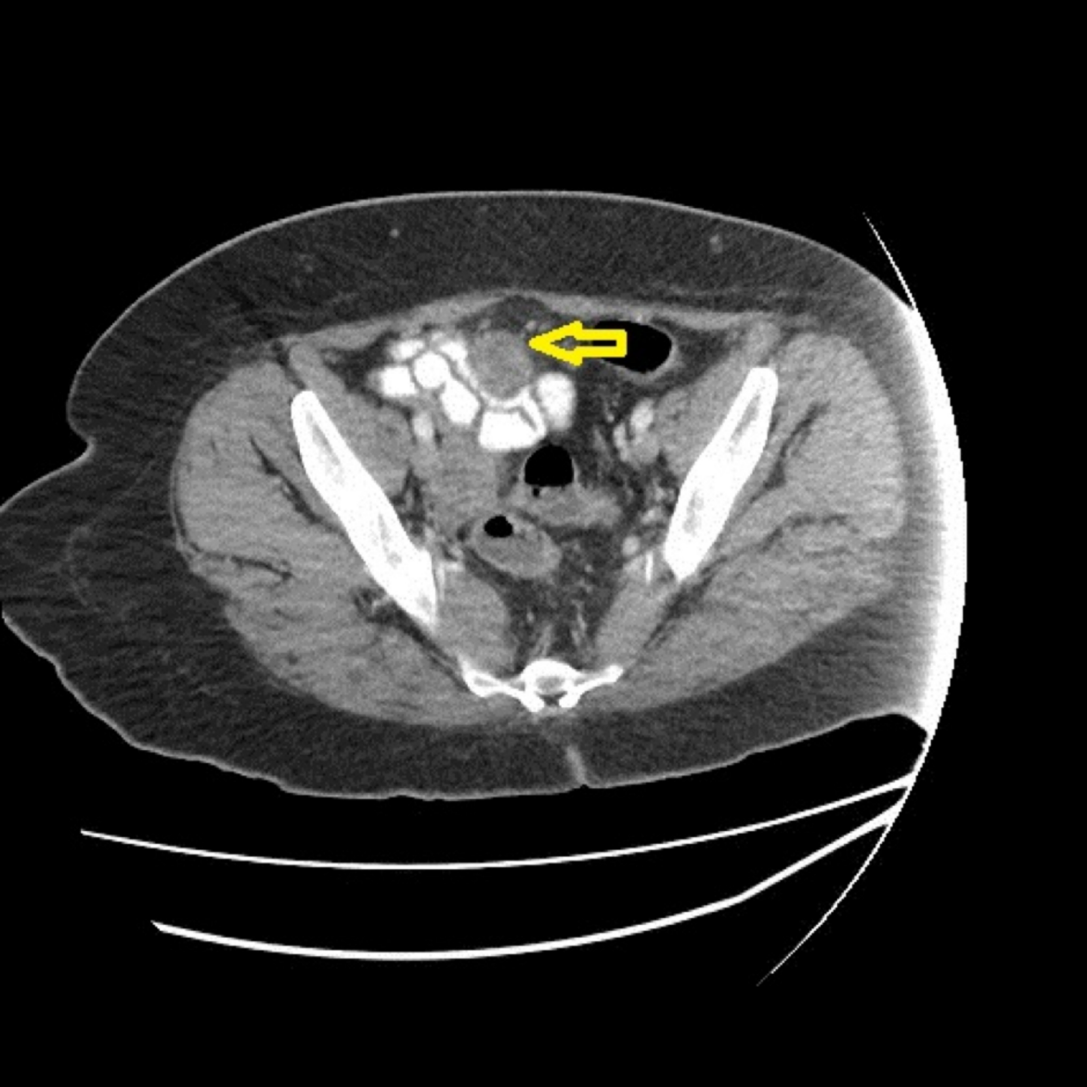
Initial CT of the abdomen and pelvis - coronal view The image shows an extensive irregular wall with luminal narrowing involving the terminal ileum with an eccentric mass as depicted by the yellow arrow CT: computed tomography

**Figure 2:**
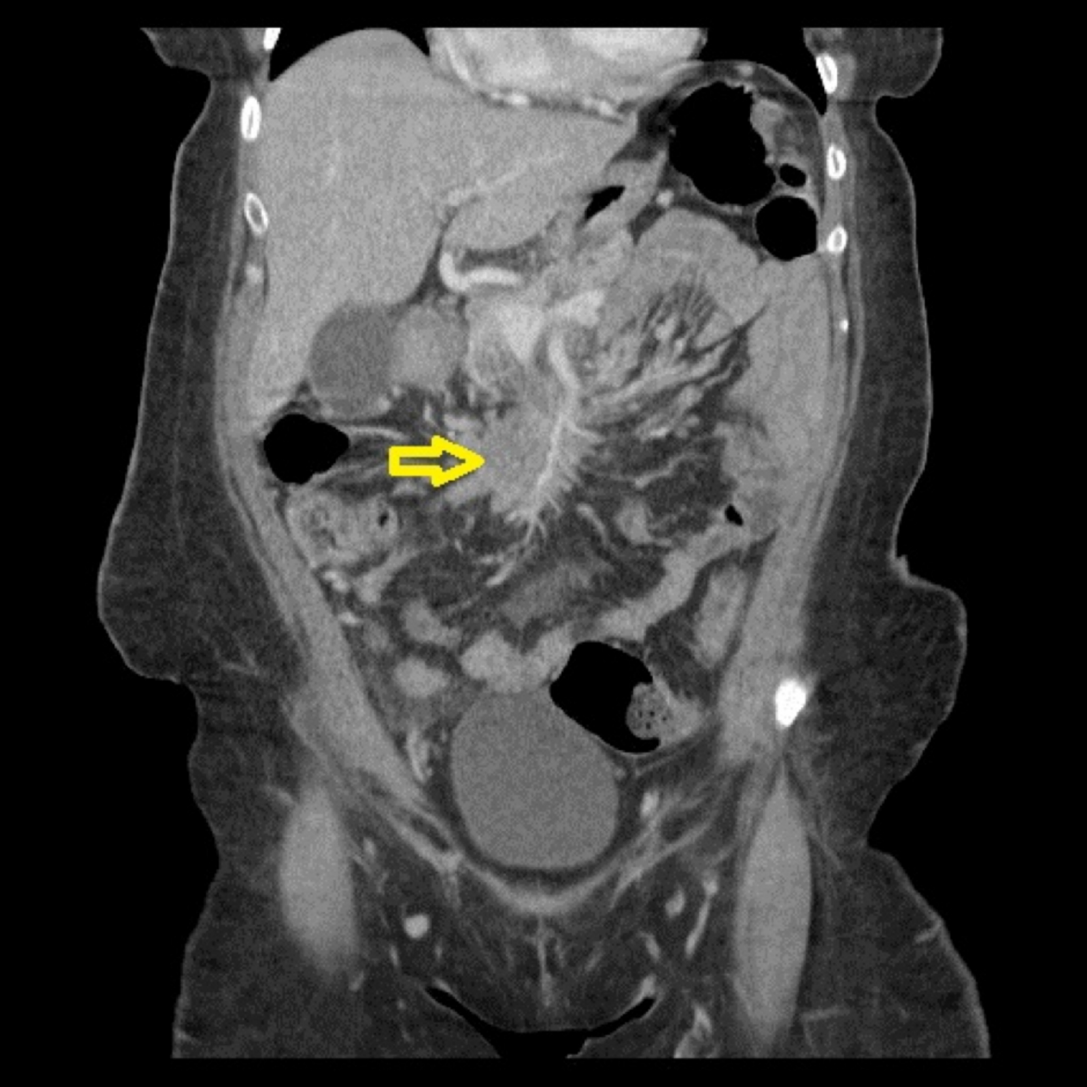
Initial CT of the abdomen and pelvis - axial view The image shows an eccentric mass and adenopathy encasing the superior mesenteric artery as depicted by the yellow arrow CT: computed tomography

A 48-year-old African American female with a past medical history of hypertension, obstructive sleep apnea, and iron deficiency anemia presented to the emergency department in early March 2017, complaining of fatigue, unintentional 40 lb weight loss for six months, and intermittent cramping and abdominal pain (rated at 9.5/10 in intensity) that interfered with sleep. The patient had an incomplete preparation of colonoscopy in early February 2017. Complete blood count (CBC) on admission showed hemoglobin of 6.8 g/dL and hematocrit of 22.5%. A CT of the abdomen with oral and intravenous contrast showed an extensive irregular wall with luminal narrowing and possible ulceration involving the terminal ileum with an eccentric mass and adenopathy along the portacaval space, one of which was encasing the superior mesenteric artery (SMA) (Figure 1, 2).

The patient was taken for an operation where a large tumor involving terminal ileum, cecum, and ascending colon with significant lymphadenopathy was found. Right hemicolectomy was performed in March 2017. Tumor markers (CEA, Ca 125, Ca 19-9) were elevated. The histological report revealed adenocarcinoma. She was found to have stage pT4aN2aM0 (stage IIIC) colon cancer with K-RAS wild type. Genetic testing performed was positive for MSH-6 and EGFR/CEP7 2.47, i.e., low copy number. She was diagnosed with Lynch syndrome in August 2017.

She was then started on the standard chemotherapy for stage III colon cancer post-resection of the primary tumor, during which the patient received a total of 12 cycles of FOLFOX. A CT of the abdomen and pelvis with contrast in September 2017 (a week after the last cycles of FOLFOX) revealed new liver metastasis along with retro-aortic and mesenteric lymph node metastasis. The patient was then switched to the FOLFIRI-bevacizumab regimen for K-RAS wild type right-sided colon cancer in October 2017. She received a total of six cycles of the treatment.

At the end of six cycles, the patient complained of increasing constipation and nausea. A CT scan was performed, and it revealed an unchanged metastatic lesion in the liver and retro-aortic and mesenteric lymph nodes. Furthermore, in that CT scan, the patient was noted to have a thrombosed superior mesenteric vein, with mesenteric mass surrounding superior mesenteric artery and vein. With these new findings, the patient's regimen was changed to FOLFIRI-cetuximab (in light of KRAS wild type), of which she received only three cycles.

**Figure 3:**
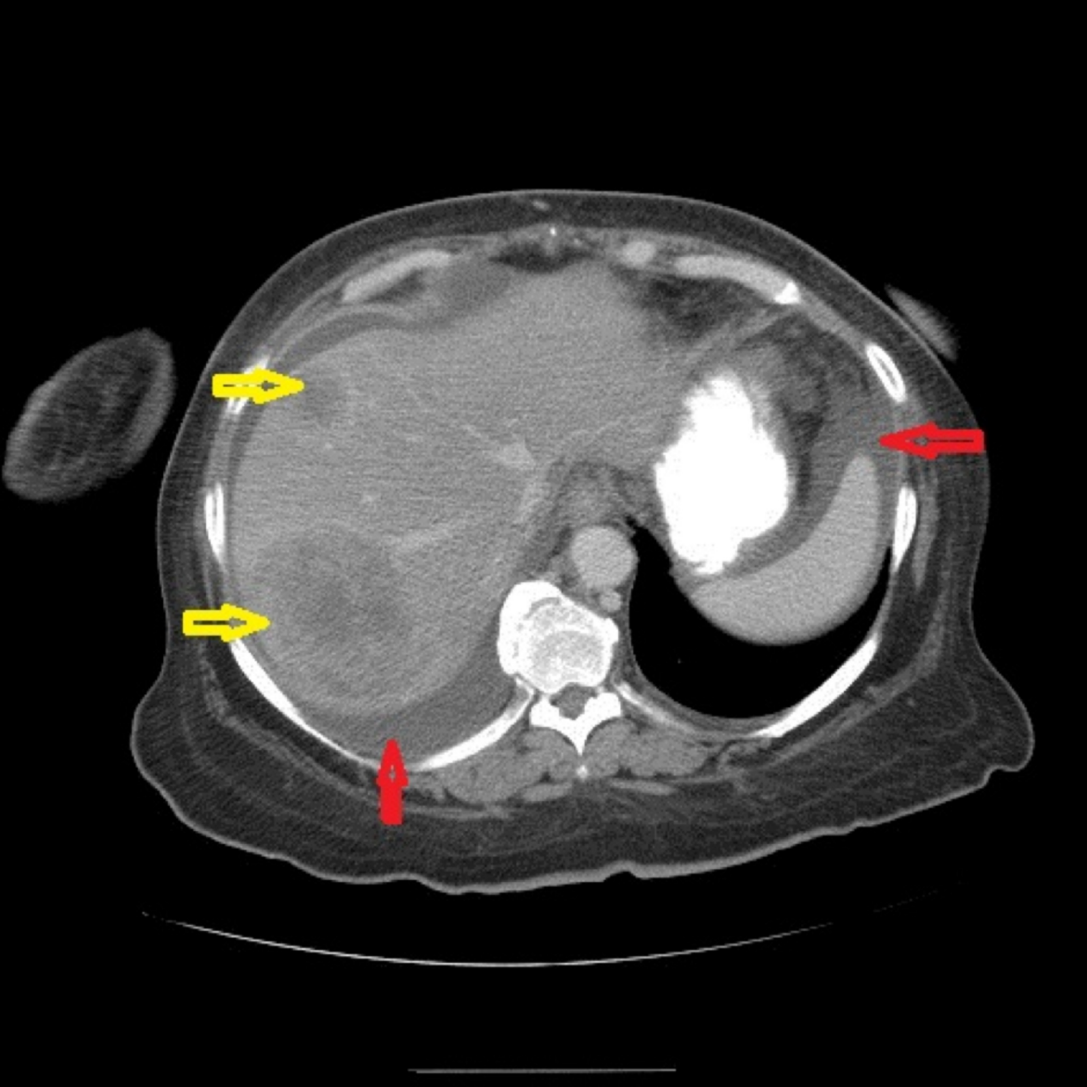
Repeat CT of the abdomen and pelvis in July 2018 - coronal view The image shows new and enlarged liver metastatic disease; the previous 48-mm metastatic lesion was later measured to be 76 x 66 mm as depicted by the yellow arrows. The red arrows show ascites CT: computed tomography

Due to the progression of her disease, the patient was started on pembrolizumab in June 2018. She received the first dose on June 8, 2018. A repeat CT scan in July 2018 showed a new and enlarged liver metastatic disease. The previous 48-mm metastatic lesion was later measured to be 76 x 66 mm along with the worsening of retroperitoneal and mesenteric lymphadenopathy (Figure 3).

**Figure 4:**
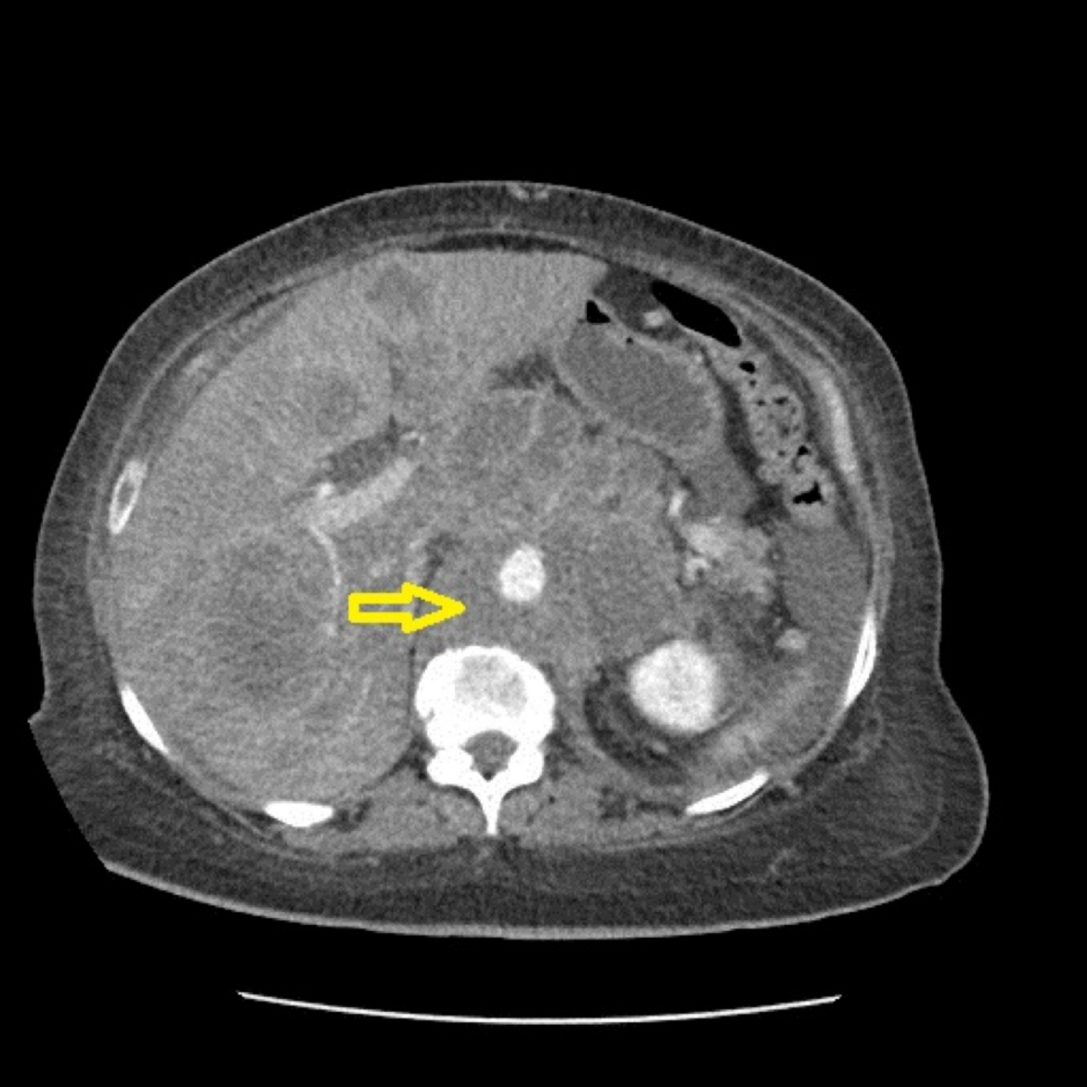
Repeat CT of the abdomen and pelvis in August 2018 - coronal view 1 The image shows multiple new and enlarged liver metastasis as well as enlarged adenopathy (yellow arrow) CT: computed tomography

**Figure 5:**
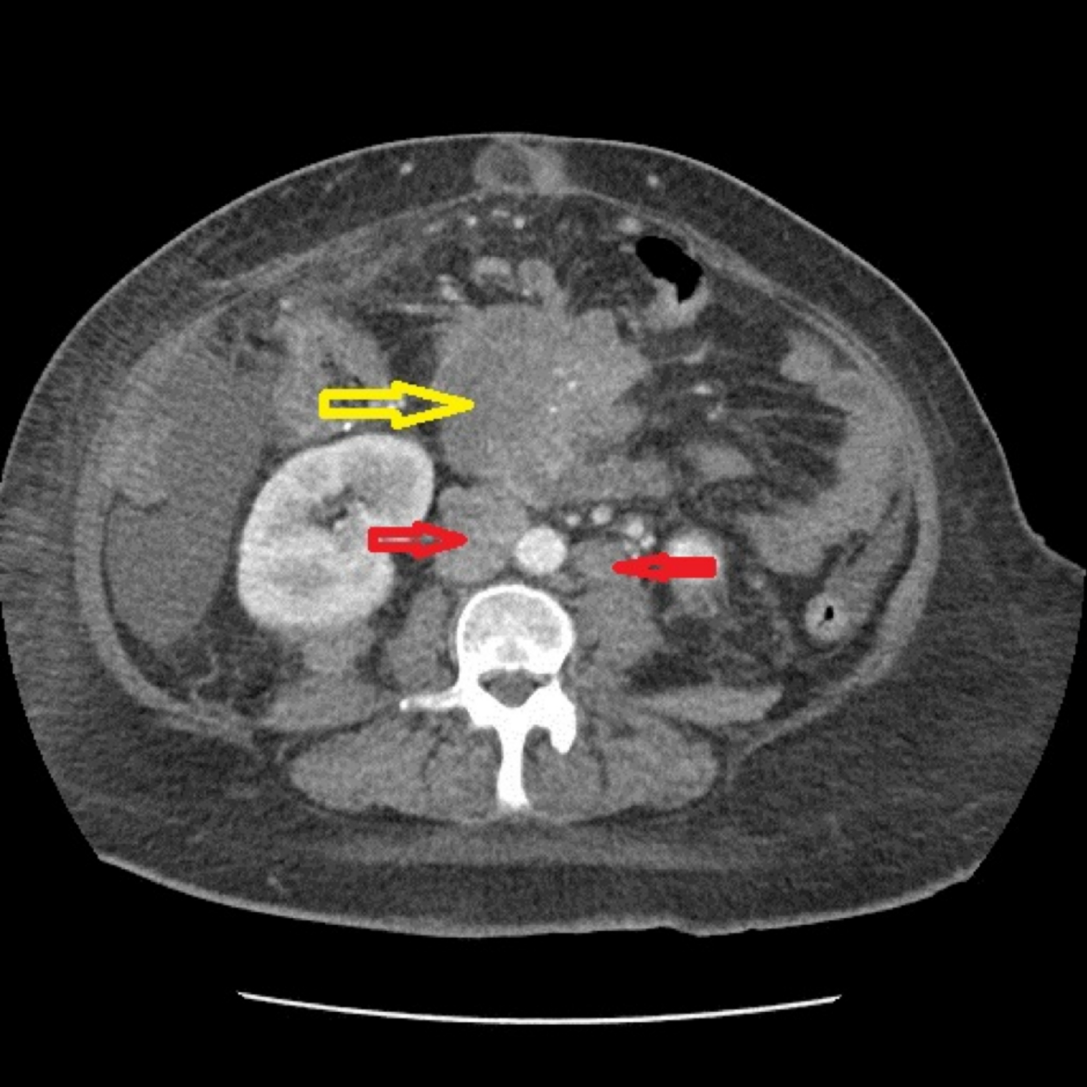
Repeat CT of the abdomen and pelvis in August 2018 - coronal view 2 The image shows an enlarged mesenteric mass (yellow arrow) and retroperitoneal adenopathy (red arrow) CT: computed tomography

It was thought to be too early to classify it as a failure of pembrolizumab treatment. Hence, the patient received two more doses with the third dose given in mid-July 2018. The patient visited the emergency department in August 2018 for intractable abdominal pain with nausea, which had been worsening for about 10 days. A CT scan revealed a new and enlarged liver metastasis as shown in Figure 4 along with enlarged mesenteric and retroperitoneal adenopathy as demonstrated in Figure 5.

In light of the advance progression of the disease, pembrolizumab was withheld, and the patient was offered palliative care. Sadly, the patient passed away about two months after being treated with pembrolizumab.

## Discussion

The emergence of ICIs in the treatment of a variety of solid tumors has transformed the landscape of cancer care. ICIs have shown a phenomenal response in a subset of patients with PD-1 expression. ICIs have also shown promising outcomes in patients with mismatch repair-deficiencies such as Lynch syndrome. Le et al. have reported improvement in immune-related objective response rate and progression-free survival in patients with mismatch repair-deficient colorectal cancer who received anti-PD1 as compared to patients with mismatch repair-proficient colorectal cancers [5]. Patients with cancers that exhibit mismatch repair-deficiencies seem to be more responsive to anti-PD1 than those whose tumors without mutations/deletions in the mismatch repair gene [5,6]. Nonetheless, in a subgroup of patients, the treatment with anti-PD1 and anti-PD-L1 may lead to rapid tumor growth known as HPD [1]. HPD is an aggressive pattern of disease with accelerating tumor growth in a significant fraction of patients on immunotherapy [1]. The definition, incidence, pathogenesis, and predictive factors of HPD are yet to be clearly established and understood. Nonetheless, numerous studies have been conducted in cancers such as those of head and neck, melanoma, and lung cancers to further define and classify HPD. Parameters such as tumor growth rate (TGR), tumor growth kinetic (TGK), and time to treatment failure (TTF) have been used to define HPD, but a universally accepted definition has not been found [1-3].

In the first study of HPD by Champiat et al., HPD was defined as a progression by Response Evaluation Criteria in Solid Tumors (RECIST) at first evaluation with a greater than or equal to a two-fold increase in TGR from baseline (before immunotherapy treatment). The study showed a 9% incidence of HPD in patients with various cancers on anti-PD-1/PD-L1. The study showed an association of HPD with higher age (those aged more than 65 years) and in patients with poor prognosis [1]. Kato et al., on the other hand, defined HPD based on three criteria: TTF of less than two months, >50% increase in tumor burden compared with pre-immunotherapy, and greater than or equal to a two-fold increase in progression pace [2]. Kato et al. used this definition to portray the genomic markers associated with HPD. Out of the 155 patients studied, all six patients with MDM2/MDM4 amplification and eight out of 10 patients with EGFR alteration showed HPD, which led to the conclusion that patients who require anti-PD1/PDL1 monotherapy should undergo genomic testing before immunotherapy [2]. Nonetheless, Ferrara et al. defined HPD as a disease progression at the first evaluation with a variation of TGR exceeding 50% before treatment and after one month of treatment. As per this definition, HPD is more common in patients with greater than two metastatic sites before the start of anti-PD-1/PD-L1 treatment. HPD is also associated with poorer prognosis in patients treated with anti-PD-1/PD-L1 [3].

Besides HPD, pseudoprogression is another distinct phenomenon observed in patients treated with anti-PD-1/PD-L1. Pseudoprogession is a unique radiological response pattern with radiologic tumor progression from baseline, which then demonstrates complete/partial resolution on subsequent radiologic assessment. Caramella et al. defined pseudoprogression as a phenomenon with initial progression in the radiological appearance of the tumors in response to ICIs, followed by a complete response, partial response, or stable disease lasting for six months or more [7]. In the case of pseudoprogression, a patient is clinically stable despite the progression of the tumor detected radiographically. The incidence of pseudoprogression was reported to be around 1.5-3.0% in solid tumors such as NSCLC and urothelial carcinoma treated with ICIs [8,9]. A recent study by Ferrara et al. reported a 4.7% incidence of pseudoprogression in patients with NSCLC treated with anti-PD-1 or anti-PD-L1 [3]. There is no consensus definition of pseudoprogression to date. In general, according to the immunotherapy response criteria recommendations, for patients on ICIs with pseudoprogression who are clinically stable, ICIs can be continued with subsequent imaging follow-up in four weeks [10].

The underlying mechanism of HPD still remains inconclusive. Xu-Monette et al. suggested that the paradoxical immunosuppression triggered by ICIs is the backbone of HPD [11]. They proposed that anti-PD-1 will act as an agonist instead of antagonist on cytotoxic-T cells, inhibit helper-T cells, and alter the PD-1 gene expression, leading to a paradoxical cytotoxic-T cells suppression and an enhancement of PD-L1/PD-1 ligation [11]. In a recent study on identifying the predictive anti-PD-1 biomarkers with mass cytometry, it was proposed that the frequency of CD14+-CD16-HLA-DRhi monocytes in peripheral blood cells prior to the commencement of therapy is the strongest predictor of response to anti-PD-1 immunotherapy [12]. Moreover, Xiong et al. further illustrated the immunogenomic landscape in patients with HPD on anti-PD-1/PD-L1 [13]. A subset of genetic alterations that includes tumor-suppressor genes, antigen-processing genes, and immune modulators as well as a subset of immune cell populations were found to be activated in HPD tumors, which may explain the underlying immunological response in these subgroups of patients presenting with HPD after starting on anti-PD-1/PD-L1 [13].

The patient described here fulfilled all the definitions proposed by Champiat et al., Kato et al., and Ferrara et al., with disease progression after the first dose of pembrolizumab [1-3]. The patient had TTF of fewer than two months, >50% increase in tumor burden prior to therapy commencement, and greater than or equal to a two-fold increase in progression pace. Prior to the commencement of anti-PD-1/PD-L1, the patient had liver metastasis, retro-aortic and mesenteric lymph node metastasis, and mesenteric mass surrounding superior mesenteric artery and vein. Ferrara et al. have reported that HPD is more common in patients with more than two metastatic sites before the start of anti-PD-1/PD-L1 [3]. Moreover, our patient’s genomic profiling showed an alteration of EGFR. Kato et al. have reported that EGFR alteration has been associated with a high risk of progression to HPD [2]. Nonetheless, we did not perform immunophenotyping or full genomic profiling for this patient. The patient was clinically deteriorating with subsequent imaging showing a continuous progression of the tumor with new metastasis, with no partial/complete resolution. The tumor growth and disease progression were so rapid and the patient ultimately passed away within two months after being started on ICIs.

To our knowledge, this report is one of the very few reports on HPD in patients with advanced colon cancer, particularly those with Lynch syndrome. Further studies are warranted to understand why some individuals benefit from immunotherapy while others do not. Knowledge of the pathogenesis and predictive factors of HPD may help clinicians to better understand the group of individuals that will likely benefit from immunotherapy. Until then, patients on immunotherapy should be closely observed for any evidence of HPD. Serial scans should be conducted and immunotherapy response criteria recommendations can be employed. If patients are found to have HPD, immunotherapy should be withheld and other treatments, such as chemoradiation, should be considered.

## Conclusions

The field of HPD is still expanding and its underlying mechanism remains elusive. Regardless, oncologists who treat patients on ICIs should be aware of the existence of HPD. Our case is one of the very few reported cases involving HPD in a metastatic colorectal cancer patient with Lynch syndrome. We hope that this will create awareness among oncologists on the occurrence of HPD, which can aid them in the management of advanced colorectal cancer patients on ICIs.
